# Using a joint triage model for multi-hospital response to a mass casualty incident in New York city

**DOI:** 10.4103/0974-2700.50746

**Published:** 2009

**Authors:** Bonnie Arquilla, Lorenzo Paladino, Charlotte Reich, Ethan Brandler, Michael Lucchesi, Sanjay Shetty

**Affiliations:** Department of Emergency Medicine, SUNY Medical Center Brooklyn, NY

**Keywords:** Hospitals, joint triage, and surge capacity

## Abstract

This paper defines a specific plan which allows two separate institutions, with different capabilities, to function as a single receiving entity in the event of a mass casualty incident. The street between the two institutions will be closed to traffic and a two-phase process initiated. Arriving ambulances will first be quickly screened to expedite the most critical patients followed by formal triage and directing patients to one of the two facilities. Preparation for this plan requires prior coordination between local authorities and the administrations of both institutions. This plan can serve as a general model for disaster preparedness when two or more institutions with different capabilities are located in close proximity.

## INTRODUCTION

Disaster preparedness has become an important issue in recent years, with most hospitals throughout the United States developing individual disaster plans. This has been the case at SUNY Health Science Center Brooklyn, where both University Hospital of Brooklyn (UHB) and Kings County Hospital Center (KCHC), located across the street from one another, have separate contingencies in the event of a mass casualty incident (MCI). The UHB is a state run tertiary care facility with a cardiac catheterization lab and cardiac surgery capabilities while KCHC is a large municipal hospital and a Level I trauma center. The former has larger decontamination facilities, while the latter has a larger total capacity. While both hospitals are affiliated with SUNY Downstate Medical School and share housestaff, they are operated independently and compete financially. Patients seen at one institution requiring specialty care at the other must be transferred in accordance with the EMTALA regulations. For example, patients presenting to UHB with major trauma must first be triaged, stabilized and then transferred across the street via ambulance, with copies of the chart and any imaging studies, for definitive care. One can imagine how cumbersome this process could become in case of any MCI. The authors of this paper set out to define an integrated response to a disaster utilizing the unique capabilities of each facility. As having affiliated institutions in close proximity to each other is common in large cities, the authors hope that the KCHC/UHB model can serve as a blueprint for other collaborative efforts during an urban MCI.

Hospitals must have triage systems to cope with potential MCIs near their facilities. Several casualties can present with little or no warning prior to initiation of city-wide emergency medical system (EMS) response plans. In addition, several casualties may be transported directly to the hospital from the incident scene by EMS vehicles, or they may simply overwhelm established EMS field triage and treatment posts and move en masse to the nearest hospital.[1] For these reasons, institutions should not rely solely on EMS to perform triage and decontamination. This is of concern because up to 80 % of disaster victims may seek hospital care without accessing EMS.[2] Recent history has borne this out: approximately 85 % of patients from the 1995 sarin gas attack in the Tokyo subway arrived with assistance from civilian motorists.[3] There is frequently a lack of communication from the scene to receiving hospitals. In numerous MCIs, initial notification was made by the first arriving casualties or ambulances.[4] This places the burden of triage and decontamination on the receiving institution.

During the World Trade Center attacks, patients who did manage to access EMS were taken either to the nearest hospitals, which were quickly overwhelmed, or to the hospitals from which the EMS crews originated, often community hospitals with no trauma capabilities.[5] Moving from the field to the front doors of the hospitals obviates the need for prehospital personnel to make designation decisions. In order to best utilize the resources and capabilities of UHB and KCHC, a joint triage plan was created which allows patients brought to a central location to be triaged depending on the severity of illness and potential need for specific resources.

## TRAFFIC FLOW

To facilitate joint triage, patients would be funneled into a central receiving location. Traffic flow routes for mass transit, ambulances, employees and the press were established in coordination with the New York City Police Department (NYPD) as well as UHB and KCHC Hospital Police. The main street between the two facilities, Clarkson Avenue (see Figure), was immediately closed and traffic diverted to streets south of the hospital. NYPD began towing vehicles remaining on the street within the hour. Lanes for ambulance triage were set up using wooden barricades. City bus routes were also diverted around the hospitals. Winthrop Ave, immediately north of Clarkson, was closed for arrival of supplies, equipment, employee parking, and for access to essential services not related to the MCI, such as dialysis. This traffic pattern was to facilitate patient flow toward the centralized triage station, while diverting press and the "convergence phenomenon" to the periphery and around the hospitals, away from the entrances.[6] Along Clarkson Avenue, there were two ambulance lanes, critical and delayed, to allow the vehicles with the more acute patients unencumbered access to the EDs.

**Figure:**
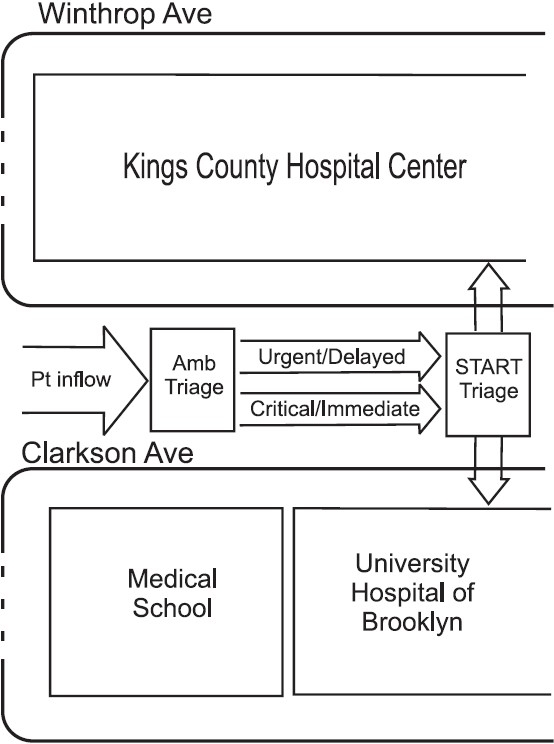
Diagram of local area of Kings County Hospital Center and University Hospital of Brooklyn showing flow of patients during an MCI utilizing the joint triage method. Amb Triage refers to Ambulance triage. START stands for Simple Triage and Rapid Treatment. North is up

## PATIENT FLOW

After receiving notification, the UHB and KCHC Command Centers would activate the joint triage. A single ambulance triage will then be established between the institutions away from the ED arrival bays, so as not to obstruct access (See Figure). Ambulances would approach from the west, stopping first in front of the medical school, where the ambulance triage officer (a senior resident or attending) would perform ambulance triage, a rapid evaluation (less than 30 seconds) consisting of identifying the type of injury (burn,crush,etc.), the anatomical location of the injury, and EMS vital signs. The ambulance triage officer would then direct ambulances designated as critical/immediate to the open lane to START triage (See Figure and below), ambulances designated urgent/delayed to the slower ambulance queue to START triage, and the least acute cases to ambulatory triage (not shown). The ambulance triage officer would proceed from vehicle to vehicle tagging each patient who would then be sent to the appropriate triage area. Ambulance triage officers would be identified by labeled vests. Strategically placed observers will monitor patient flow.

The next step would combine Simple Triage and Rapid Treatment (START) triage with directing patients to either of the two facilities. START triage would be located at a designated point in the street equidistant from the Emergency Departments (EDs) of UHB and KCHC. Waterproof signs for key areas will be posted to help funnel patients into the central triage area and prevent potentially contaminated patients from breeching the integrity of either institution. Two decontamination tents, each in front of its respective institution, would be deployed. The KCHC tent holds fewer patients but can accommodate stretchers, while the UHB tent is designed for a higher volume of ambulatory patients. In general, multi-trauma patients will be sent to KCHC, and less urgent or ambulatory patients will be sent to UHB depending on the ability of each institution to manage patient influx.

The standard four color triage categories would be used: red for immediate, yellow for urgent, green for minor injuries (the so-called “walking wounded”), and black for the deceased/expectant. Separate treatment areas would be designated for each category. Triage tags are made with three copies, one for the patient, one for the triage officer, and a third for the receiving institution. Recorders (a clerk, medical student, etc.) assigned to each START triage officer will keep track of names, total number of ambulance patients, and number of patients triaged to each institution. START triage officers will also have radios to communicate with the Command Centers, so that resources may be shifted appropriately based on the evolving need. Though START triage has come under attack in recent years, a retrospective review of 1144 trauma patients triaged by different algorithms suggests that START triage is still a relatively sensitive tool for predicting critical injuries.[7] There is much room for improvement, however, and disaster triage remains an active area of investigation.

A single ambulatory triage station will be set up between UHB and KCHC. The KCHC will provide four nurses and one attending physician for ambulatory triage, UHB will provide an attendant, a nurse, and a technician with resuscitation equipment. Because triage is a dynamic process, with patients' conditions changing while they await their designated level of care, it often becomes necessary to up or down triage patients accordingly. For this reason, stretchers and wheelchairs will be kept by ambulatory triage in the event that one of the walking wounded deteriorates and requires transfer to the next level of care.

## COMMUNICATION

Even the most comprehensive disaster plan requires real time communication amongst the participants to be effective. It has been well documented that failure of the primary mode of communication is the rule during MCIs. For this reason, we have employed the use of Nextel mobile phones in the event of an MCI. These phones are used for horizontal communication with the EDs, the triage sites, and with the trauma and perioperative services. The phones are used as walkie-talkies, with multiple personnel communicating over a single frequency. Vertical communication with the Command Centers is via a single disaster management officer in either ED. This person can either be a senior attending or nurse administrator, depending on staffing. To ensure that these phones are always ready for use, we built an easily accessible secure cabinet adjacent to the ambulance entrance, to allow continuous charging of the phones. The phones are checked biweekly to ensure proper functioning. The Nextel seems to be the most reliable communications device at this time. As a backup, the NYPD will provide 10 radios on a separate channel, with medical students serving as runners when required.

## SUMMARY

Difficulties in MCIs usually arise not from a lack of resources, but from a failure to coordinate them.[4] This paper attempts to define a plan, which is being specifically designed to allow for the best allocation of the resources available to both UHB and KCHC; remains applicable to other institutions and maintains the flexibility necessary to deal with all possible disaster scenarios in this era of evolving global threat.
